# Control Measures for Severe Acute Respiratory Syndrome (SARS) in Taiwan

**DOI:** 10.3201/eid0906.030283

**Published:** 2003-06

**Authors:** Shiing-Jer Twu, Tzay-Jinn Chen, Chien-Jen Chen, Sonja J. Olsen, Long-Teng Lee, Tamara Fisk, Kwo-Hsiung Hsu, Shan-Chwen Chang, Kow-Tong Chen, I-Hsin Chiang, Yi-Chun Wu, Jiunn-Shyan Wu, Scott F. Dowell

**Affiliations:** * Taiwan Department of Health, Taipei, Taiwan;; † International Emerging Infections Program, Centers for Disease Control and Prevention, Bangkok, Thailand; ‡ Emory University School of Medicine, Atlanta, Georgia, USA; § National Taiwan University, Taipei, Taiwan

**Keywords:** SARS, pneumonia, prevention, Taiwan, dispatch

## Abstract

As of April 14, 2003, Taiwan had had 23 probable cases of severe acute respiratory syndrome (SARS), all imported. Taiwan isolated these first 23 patients with probable SARS in negative-pressure rooms; extensive personal protective equipment was used for healthcare workers and visitors. For the first 6 weeks of the SARS outbreak, recognized spread was limited to one healthcare worker and three household contacts.

The global spread of severe acute respiratory syndrome (SARS) has proceeded with unprecedented speed, overwhelming many hospitals and some public health systems in a matter of weeks. As of April 14, 2003, a total of 3,169 cases had been reported from more than 20 countries. In many locations, the introduction of the disease by ill travelers has soon been followed by spread to healthcare workers and household contacts. In the most mature outbreaks, in Hong Kong and Hanoi, 46% and 63% of cases, respectively, were reported in healthcare workers, and hospital spread has also characterized the larger outbreaks in Singapore and Toronto (*1*,*2*).

Taiwan, with its close proximity to the epicenters of severe acute respiratory syndrome (SARS) in Guangdong Province and Hong Kong and its extensive business and cultural ties, has heavy travel volume from the most affected areas. The first probable SARS case-patient in Taiwan returned from Guangdong and Hong Kong early in the global outbreak, on February 21, 2003, and a series of other importations have been documented since that time. Factors that contribute to spread of infection in a given location are not well understood but may include not only the number of coronavirus-infected persons but also whether any of these persons are particularly infectious, whether they are identified early in their illness, and how effectively they are isolated. To contribute to discussions on how to effectively prevent transmission, we believe reporting the early experience with limited spread of the disease in Taiwan, along with a thorough description of the control measures taken, is important.

## Epidemiology of SARS in Taiwan

The first recognized SARS patient in Taiwan was in a 54-year-old businessman who traveled to Guangdong Province, China, on February 5, 2003, and returned to Taipei by way of Hong Kong on February 21. On February 25, fever and myalgia, and later a dry cough, developed, but he was not hospitalized until March 8. Several hours after admission, he was intubated and required mechanical ventilation for 13 days. During the initial hospitalization, he was cared for in a single intensive care unit (ICU) room by healthcare workers who used standard nursing (universal) precautions. When pneumonia was diagnosed in the patient’s wife on the morning of March 14, both patients were placed in isolation rooms; by the afternoon they were isolated in ICU negative-pressure rooms with full precautions, as described below. Fever developed in their son on March 17, followed by cough on March 20; he was hospitalized in a negative-pressure isolation room on March 21. The wife and son were exposed during the period before full protective measures were in place, and SARS developed in both. Both required mechanical ventilation. The illnesses in the wife and son were confirmed by reverse-transcription–polymerase chain reaction (RT-PCR) testing to be associated with the novel SARS coronavirus (*3*–*5*).

As of April 14, 23 persons in Taiwan met the World Health Organization (WHO) criteria for a probable case of SARS. Of these, eight had SARS-associated coronavirus identified in throat swabs by PCR. An additional 120 reports of possible case-patients with compatible travel or contact were investigated, and 13 remained under investigation. Of the probable case-patients, 19 (83%) reported travel to mainland China and Hong Kong in the 10 days before illness onset, and 4 represented secondary spread. The patients with secondary cases included the two family members described above, a person who acquired it in his household from a Hong Kong visitor (representing 13% of cases), and a single healthcare worker (representing 4% of cases).

The single case in a healthcare worker was in a 32-year-old physician who cared for the wife of the initial case-patient. On March 14, the physician had performed a chest ultrasound that lasted approximately 30 minutes; he spent an additional hour in the room on March 17 during and after intubation. He was at the side of the bed supervising the intubation and in a direct line of droplet spread when the patient had episodes of coughing, sometimes partially sitting up. The physician reported wearing an N95 mask, eyeglasses without goggles, two pairs of gloves, and two gowns. His illness began was on March 21, with clinical features that met the criteria for a probable SARS case and laboratory confirmation of coronavirus infection by RT-PCR. None of the other five persons present in the room for the intubation became ill after 28 days of follow-up.

## SARS Control Measures in Taiwan

Beginning with the recognition of the first SARS case on March 14, Taiwan moved aggressively to isolate all suspected or probable case-patients in negative-pressure rooms and to equip all healthcare workers with enhanced protective equipment. Assistance from the U.S. Centers for Disease Control and Prevention (CDC) was requested, and a team has worked with Taiwan Center for Disease Control officials since March 16 to implement a framework for SARS control.

In March through April, 2003, Taiwan had a total of 764 negative-pressure rooms. Most were specially designed isolation rooms with HEPA-filtered air, negative pressure under continuous electronic monitoring, separate bathroom, and anteroom so that two doors separate the SARS patient from the rest of the hospital. The Center for Disease Control in Taiwan tracked available isolation rooms on an ongoing basis, and all of the first 23 probable case-patients were cared for in such negative-pressure rooms, located in 15 hospitals across the island. Resources in the highly affected areas of Hong Kong, Singapore, and Toronto may have been quite similar or superior to those in Taiwan. In contrast, neither of the two hospitals treating SARS patients in Hanoi had negative-pressure rooms, and such rooms were not commonly available in hospitals in Thailand, Laos, or other Asian countries (T. Uyeki, M. Simmerman, pers. comm.).

A team from the Taiwan Center for Disease Control visited each of the 15 hospitals caring for SARS patients to audit and implement strict infection control practices. Widespread education of healthcare workers on SARS control, with written guidelines, pictures, and demonstrations, was undertaken. Full protective equipment for healthcare workers was also widely available, and such equipment was provided and monitored under the conservative assumption, made early in the Taiwan outbreak, that airborne or fomite transmission of the agent should be presumed until proven otherwise. In addition to the handwashing and barrier precautions with gown, gloves, mask, and eye protection recommended by WHO, most healthcare workers in Taiwan used a disposable second layer of protective clothing (outer gloves, outer gown, head and foot covering) that were discarded before workers left the anteroom to prevent fomite transmission to other areas. Surgical or loose-fitting masks were actively discouraged, and N95 or greater filtration masks with tight-fitting seals were uniformly available. These precautions were used for 354 of 370 patient-care-days for the first 23 patients through April 14. After SARS was identified in the physician, use of goggles rather than glasses and careful fitting of masks were reemphasized to hospital infection control departments. Visits by family members were prohibited or minimized.

Active surveillance of exposed healthcare workers and contacts of patients was begun. In addition, the infection control nurses at the 15 hospitals passively monitored absenteeism in all employees. As of April 14, no other suspected or probable cases of SARS were identified through active daily monitoring of 525 healthcare workers, 210 work colleagues, 54 family and friends, and 31 public health staff who were exposed to the first 20 case-patients. Although some healthcare workers and household contacts of discharged patients had ongoing contact with SARS cases, as of this writing the outer limit of the incubation period (14 days) had already passed for contacts of 15 of the 23 cases, including 45 family and 220 work contacts. A more thorough epidemiologic investigation of close contacts was under way to determine the risk for transmission by level of contact and protection.

Beginning March 28, contacts of known patients, both suspected and probable, were quarantined at home for 10 days. As of April 14, a total of 1,572 persons had been put on home quarantine. This included healthcare workers exposed outside isolation settings, family and other close contacts, and those on airplanes with ill SARS patients if seated within three rows in front of or three rows behind a patient. Fourteen medical personnel were quarantined because they had had contact with two probable case-patients before they were hospitalized, including six present during endoscopy of one of the patients.

## Conclusions

Approximately a 2-week interval elapsed between recognition of SARS outbreaks in Hanoi and Hong Kong and the introduction of SARS in Taiwan, giving clinicians and public health authorities in Taiwan some opportunity to act with more knowledge of its infectivity and severity. Available evidence suggests that the spread of SARS within Taiwan from the known imported cases was limited during the first 6 weeks after importation began. As occurred in some other SARS-affected areas, Taiwan initiated a strategy of aggressive public health measures combined with stringent hospital infection control practices that met, and in some instances exceeded, those recommended by WHO or CDC.

As of April 14, the SARS epidemic in Taiwan was at an early stage. Hospitals in Taiwan were able to care for all SARS patients in individual negative-pressure rooms; cohorting of patients in group wards was not necessary, and hospital staffing levels were not strained, as they have been in some cities with far more SARS patients. Also, although surveillance for healthcare worker transmission appeared thorough, other episodes of spread may have been missed. Concern about stigmatization and quarantine may have resulted in some concealment of illness not requiring hospitalization, and persons with mild illness or subclinical infection would not have been isolated. In part because of the challenge of ascertaining the true extent of transmission, health departments elsewhere have believed the SARS epidemics to be under control, only to find later that spread had occurred beyond what was recognized.

Taiwan continues to identify new cases in patients who report recent travel to mainland China and other SARS-endemic areas. Each year, more than 4 million visits are made by Taiwanese to mainland China for business and tourism (Bureau of Immigration, Ministry of the Interior, unpub. data), and the vast majority of all flights return through Hong Kong. Although such travel is being curtailed, Taiwan clearly will be at ongoing risk for importation and spread of SARS. Indeed, an apparent nosocomial cluster in Taipei was subsequently reported and remains under active investigation. The appearance of this cluster is sobering and underscores the fragility of SARS control measures in the setting of ongoing international spread of the disease.
